# What the Papers Say

**DOI:** 10.1093/jhps/hnv066

**Published:** 2015-10-26

**Authors:** Ajay Malviya

**Affiliations:** Northumbria Healthcare NHS Foundation Trust, Newcastle upon Tyne, NE12 8EW

## Abstract

The *Journal of Hip Preservation Surgery* (*JHPS*) is not the only place where work in the field of hip preservation may be published. Although our aim is to offer the best of the best, we continue to be fascinated by work that finds it way into journals other than our own. There is much to learn from it so *JHPS* has selected six recent and topical articles for those who seek a brief summary of what is taking place in our ever-fascinating world of hip preservation. What you see here are the mildly edited abstracts of the original articles, to give them what *JHPS* hopes is a more readable feel. If you are pushed for time, what follows should take you no more than 10 min to read. So here goes …

## THREE-DIMENSIONAL CT SCAN FOR QUANTITATIVE EVALUATION OF RESIDUAL PINCER LESIONS AFTER ARTHROSCOPIC SURGERY FOR FEMOROACETABULAR IMPINGEMENT

Researchers from Beijing, China have looked to determine the clinical characteristics of residual bony impingement lesions after arthroscopic treatment for pincer-type femoroacetabular impingement (FAI) and its effect on the clinical outcome after 2 years of follow-up [1].

The study includes 30 patients who underwent arthroscopic surgery for symptomatic pincer-type FAI at a mean age of 34.5 years and mean follow-up of 26.3 months. Clinical outcomes were evaluated using the modified Harris Hip Score (mHHS) and satisfaction scores. Three-dimensional Computer Tomography (CT) scan was used to quantitatively assess the incidence and amount (residual rate) of residual acetabular bony impingement. According to residual rates, the patients were divided into three groups: group 1, residual rate <10%; group 2, residual rate 10–20 % and group 3, residual rate >20 %.

Nineteen patients (63.3%) had residual bony impingement lesions after surgery; 16 of who (84.2 %) had residual bony impingement lesions posterior to the actual acetabular resection zones. The preoperative and post-operative bony impingement angles were 77.47° (CI ± 21.31°) and 12.94° (CI ± 18.04°), respectively. The mean residual rate was 14.48%. The overall mHHS significantly improved (*P* < 0.001) from 55.2 preoperatively to 94.7 post-operatively. The overall satisfaction rate was 76.7%. The post-operative mHHS of groups 1–3 were 95.9, 95.2 and 85.5 respectively; with group 3 having significantly lower post-operative mHHS compared with the other two groups (*P* = 0.001). The satisfaction rates in groups 1–3 were 92.9, 80 and 33.3 % respectively; with group 3 again performing significantly worse than the other two groups (*P* = 0.017). There was a significant inverse linear relationship between the residual rate of bony impingement lesions and the post-operative mHHS (*R*^2 ^= 0.516, *P* < 0.05).

The authors therefore concluded that the incidence of residual impingement lesions after arthroscopic was high (63.3%), with patients having residual impingement of more than 20% exhibiting significantly lower clinical and satisfaction rates.

## RANDOMIZED CONTROLLED TRIAL FOR THE USE OF PLATELET RICH PLASMA AFTER HIP ARTRHROSCOPIC SURGERY

A randomized control trial from Santiago, Chile has uniquely evaluated the clinical and immunologic effects of intra-articular platelet-rich plasma (PRP) after arthroscopic hip surgery for FAI [2].

Patients were randomized either to receive an intra-articular injection of PRP (group I, *n* = 30) or nothing (group II, *n* = 27) at the end of hip arthroscopic surgery.

Clinical outcome was measures using the mHHS at 3, 6 and 24 months after surgery. Pain was evaluated using a visual analog scale 24 h, 48 h, 3 months and 6 months after surgery. The radiologic outcome was analysed using radiographs and magnetic resonance imaging (MRI) obtained before surgery and 6 months after surgery. Labral integration and joint effusion were evaluated with MRI at 6 months.

The visual analog scale score 48 h after surgery was significantly better for the PRP group as compared with the control (3.04 versus 5.28; *P* < 0.05). At the 3-month and 24-month follow-up, the mHHS was similar in both groups with no significant difference. The 6-month follow-up MRI showed no effusion in 36.7% of patients in the PRP group compared with 21.1% of patients in the control group (*P* = 0.013). Labral integration was similar in both groups (*P* = 0.76).

In this randomized study, PRP resulted in lower post-operative pain scores at 48 h and fewer joint effusions at 6 months. These findings suggest that PRP may have a beneficial effect by reducing post-operative inflammation without influencing the outcome scores in the long term.

## WHICH CLASSIFICATION SYSTEM SHOULD WE USE FOR GRADING CHONDRAL LESIONS?

A multi-centered international collaborative effort between Chile, Saudi Arabia and Australia reported the interobserver and intraobserver reliability of three chondral damage classifications (Outerbridge, Beck and Haddad) used to assess articular cartilage damage during hip arthroscopy [3].

This prospective study included all patients who underwent hip arthroscopy for FAI with evidence of chondral damage at the time of surgery. Intra-articular recordings were obtained during the operation in a standardized way from two different hospitals in two countries by three different surgeons. Four experienced surgeons independently analysed the recordings twice in randomized order and 4 months apart and classified the lesions according to the Outerbridge, Beck and Haddad classifications of chondral damage. The values obtained were used for interobserver and intraobserver analysis. Percentage of agreement and weighted Cohen κ values were calculated.

Absolute agreement between observers was present in 12.5% of the cases for the Outerbridge classification, in 20% of the cases for the Beck classification, and in 40% of the cases for the Haddad classification. For interobserver reliability, the average weighted Cohen κ values were 0.28, 0.33 and 0.47 for the Outerbridge, Beck and Haddad classification systems, respectively. For intraobserver reliability, the mean Cohen κ values were 0.62, 0.63 and 0.68 for the Outerbridge, Beck, and Haddad classification systems, respectively.

The authors concluded that the Haddad classification had the best interobserver reliability with no difference in the intraobserver reliability among the three classifications studied.

## AUTOLOGOUS MATRIX-INDUCED CHONDROGENESIS FOR CHONDRAL LESIONS IN THE HIP

Management of high-grade chondral lesion during hip arthroscopy can be difficult. Italian investigators have compared the results of microfracture (MFx) with a technique of enhanced microfracture autologous matrix-induced chondrogenesis (AMIC) in a single-centre retrospective analysis of a consecutive series of patients of 147 patients [4].

Acetabular grade III and IV chondral lesions measuring between 2 and 8 cm^2^ were treated by MFx in 77 and AMIC in 70 patients. The outcome was assessed using the mHHS at 6 months and 1–5 years post-operatively. The outcome in both groups was significantly improved at 6 months and 1 year post-operatively. During the subsequent 4 years the outcome in the MFx group slowly deteriorated, whereas that in the AMIC group remained stable. Six patients in the MFx group subsequently required total hip arthroplasty, compared with none in the AMIC group. The authors concluded that the short-term clinical outcome improves in patients with acetabular chondral damage following both MFx and AMIC; however, the AMIC group had better and more durable improvement, particularly in patients with large (≥ 4 cm^2^) lesions.

## REDUCING THE RISK OF NERVE INJURY DURING BERNESE PERIACETABULAR OSTEOTOMY

Another international collaboration, on this occasion between Iran and Switzerland, looked at precautions to reduce the risk of nerve injury during periacetabular osteotomy [5].

In this cadaveric study the modified Smith-Petersen and Kocher-Langenbeck approaches were used to expose the lateral cutaneous nerve of the thigh and the femoral, obturator and sciatic nerves in order to study the risk of injury to these structures during the dissection, osteotomy and acetabular reorientation stages of a Bernese peri-acetabular osteotomy.

Injury of the lateral cutaneous nerve of thigh was less likely to occur if an osteotomy of the anterior superior iliac spine had been carried out before exposing the hip. The obturator nerve was likely to be injured during unprotected osteotomy of the pubis if the far cortex was penetrated by more than 5 mm. This could be avoided by inclining the osteotome 45° medially and performing the osteotomy at least 2 cm medial to the iliopectineal eminence. The sciatic nerve could be injured during the first and last stages of the osteotomy if the osteotome perforated the lateral cortex of ischium and the ilio-ischial junction by more than 10 mm. The femoral nerve could be stretched or entrapped during osteotomy of the pubis if there was significant rotational or linear displacement of the acetabulum. Anterior or medial displacement of < 2 cm and lateral tilt (retroversion) of <30° were safe margins. The combination of retroversion and anterior displacement could increase tension on the nerve.

The authors have made useful recommendations with regards observation of anatomical details, proper handling of the osteotomes and careful manipulation of the acetabular fragment to reduce the neurological complications of Bernese periacetabular osteotomy.

## DOES PERIACETABULAR OSTEOTOMY FOR HIP DYSPLASIA MODULATE CARTILAGE BIOCHEMISTRY?

Continuing with the theme of multinational collaborative studies, researchers in Boston, United States and Bern, Switzerland looked at cartilage biochemistry after periacetabular osteotomy. The study tried to determine whether the proteoglycan content, as measured with delayed gadolinium-enhanced MRI of cartilage (dGEMRIC), could be modulated with the alteration of the hip joint biomechanics after such osteotomy [6].

In this prospective cohort study, 37 patients with no or minimal osteoarthritis were treated with periacetabular osteotomy for symptomatic acetabular dysplasia. All patients had preoperative and 1-year follow-up dGEMRIC scans. Twenty-eight of the 37 also had 2-year scans. The changes in dGEMRIC findings and hip morphology between the preoperative visit and the examinations at 1 and 2 years following the periacetabular osteotomy were assessed.

The mean preoperative dGEMRIC index was 561.6 ms; this decreased to 515.2 ms at 1 year after periacetabular osteotomy but subsequently recovered to 529.2 ms at 2 years post-operatively. The decrease in the dGEMRIC index of the acetabular cartilage after surgery was most apparent at the superior aspect of the acetabulum, where the decrease in mechanical loading after periacetabular osteotomy would be most pronounced. All domains of the Western Ontario and McMaster Universities Osteoarthritis Index demonstrated significant improvement from the preoperative to the post-operative visits (all *P* < 0.001).

The authors concluded that periacetabular osteotomy for developmental dysplasia of the hip alters the mechanical loading of articular cartilage in the hip, which in turn would alter the cartilage matrix composition, as demonstrated by dGEMRIC.
